# Supplementary material for: Are carbon emissions trading and green financial instruments synergistic? -Comprehensive quantitative research based on content analysis
Source: PLoS One. 2024 Mar 7;19(3):e0298601. doi: 10.1371/journal.pone.0298601 (PMC10919601; doi:10.1371/journal.pone.0298601)
Supplement: S1 Data — (ZIP) [file pone.0298601.s001.zip › Data/地级市Z_F工作报告.zip!/║╙─╧/╫ñ┬φ╡Ω╩╨/└┤╫╘╣1⁄2╓┌║┼í╛┬φ┐╦╩2╛▌═°í┐_2005.docx]

各位代表:

现在，我代表市人民政府，向大会作工作报告，请予审议，并请各位政协委员提出意见。

折叠编辑本段2004年政府工作回顾

2004年，市人民政府在省委、省政府和市委的领导下，在市人大、市政协的监督和支持下，团结带领全市人民，坚持以经济建设为中心，深入开展"三项建设"活动，较好地完成了市一届人大五次会议确定的各项任务。初步核算，全市完成生产总值444.7亿元，增长14.6%。其中第一产业增加值完成157亿元，增长28.4%;第二产业增加值完成173.2亿元，增长10.1%;第三产业增加值完成114.5亿元，增长8.5%。

(一)项目建设成效显著，固定资产投资快速增长。深入开展"项目建设年"活动，全市投资项目建设力度加大，年初确定的71个重点项目完成投资51.42亿元，较上年增长31.98%。其中计划竣工项目完成投资20.99亿元，续建项目完成投资16.06亿元，计划新开工项目完成投资14.37亿元，前期项目进展基本顺利。蓝天集团西气东输豫南支线及年产10万吨甲醇、天方药业2000m3螺旋霉素碱、华骏车辆铸造区迁建、华中正大金霉素扩建、骏马化工年产60万吨高浓度复合肥和商桐路、明临路等16个项目相继竣工;豫龙水泥日产5000吨熟料生产线、白云纸业年产10万吨填平补齐和11条县乡公路等项目进入扫尾;蓝天集团30万吨甲醇、骏马化工年产3万吨三聚氰胺、小洪河治理工程等20个续建项目进展顺利。重点项目实施拉动了投资的快速增长，全社会固定资产投资完成116.4亿元，增长34.4%，其中城镇以上固定资产投资70.8亿元，增长48.4%。

(二)结构调整稳步推进，产业结构继续优化。农业结构得到优化,优质小麦面积发展到617万亩,占小麦播种面积的69.1%;优质玉米、花生及蔬菜、花木、食用菌等种植面积扩大，农业区域化特点更加鲜明。粮食生产呈恢复性快速增长，总产达539.7万吨，增长61.1%。畜牧业增加值达到66.2亿元，增长6.6%，肉、蛋、奶总产量分别增长6.2%、8%和31.9%。全年完成成片造林24.42万亩，新建完善农田林网510万亩，通道绿化2573公里，正阳、遂平、汝南、新蔡通过平原绿化高级达标验收。工业结构得到改善，效益稳步提高，制药、化肥、水泥等主导产品生产规模扩大，重点行业、骨干企业拉动作用增强;高新技术产业增加值完成4亿元，增长30.4%;限额以上工业完成增加值56亿元，增长18%，实现利税14.9亿元，增长17.2%;综合效益指数为133.41%，同比提高6.1个百分点。第三产业发展势头良好，增加值及增幅均有较大提高;社会消费品零售总额完成146.9亿元，增长15.1%;实现旅游收入2.35亿元，增长28.7%;金融保险、邮电通讯、房地产、餐饮、商贸等服务业发展水平提高。非公有制经济稳步发展，实现增加值203亿元，同比增长10.3%，占全市生产总值的45.7%。

(三)城镇建设继续推进，城镇化进程步伐加快。全年完成城市建设总投资6.7亿元，增长5.8%。金色置地广场一期、温州步行街、风光路改造、南海路北段和健康路西段等工程相继竣工;前进路改造、练江路拓宽、新一代天气雷达、会展中心等项目进展顺利;新区建设规划及中心区建设布局方案已经完成;城区绿化取得新的成效，绿化覆盖率达到23.82%。深入开展环境综合整治活动，市容市貌发生了新的变化。县城和小城镇建设步伐加快，城镇化水平达到16.76%。

(四)改革开放迈出新步伐，外经外贸取得新成绩。深化农村税费改革，全面落实农业税降点和直补政策，农民实际减负增收4.23亿元;国企改革继续推进，全市140户拟改制工业企业有130户完成改制任务，占拟改制企业总数的94%;粮食流通体制改革稳步实施，农村信用社改革全面展开。对外开放取得新进展，共落实东西合作项目383个、资金24.2亿元。积极开展对外贸易，完成出口创汇4551万美元，增长41.1%;实际利用外资2438万美元，增长61%。

(五)就业和社会保障力度加大，人民生活进一步改善。城镇新增就业人员39464人，下岗失业人员累计实现再就业17624人，其中"4050"人员实现再就业7062人;城镇登记失业率3.1%;发放下岗职工基本生活费1267.8万元、企业离退休人员养老金23200万元，按时足额发放率100%;为99292人发放低保金6485万元，做到了应保尽保。扶贫开发成效明显，全年巩固温饱人口11万人，解决温饱人口7万人。全市城镇居民人均可支配收入达到6179元，增长8.9%，农民人均纯收入2226元，增长29%。城乡居民储蓄存款余额279.5亿元，增长19.5%。全市地方财政一般预算收入完成12.8亿元，增长20%，支出完成40亿元，增长20.9%。

(六)精神文明和民主法制建设得到加强，社会大局稳定。认真实践"三个代表"重要思想，深入开展精神文明创建活动，城乡面貌和干部群众的精神风貌发生了新的变化。坚持依法治市，开展普法教育，增强了全市人民的法制观念，提高了依法行政水平。自觉接受人大法律监督和政协民主监督，认真办理人大代表建议和政协提案。贯彻落实行政许可法，积极推进政务公开，取消行政许可项目51项。深入开展"平安建设年"活动，加强社会治安综合治理，严厉打击各种刑事犯罪，全市刑事案件和可防案件发案率同比分别下降41.4%和43.8%，命案破案率达到96.37%，被评为全省社会治安综合治理先进市。高度重视安全生产工作，全面落实安全生产责任制，全年没有发生重大安全事故。进一步加强信访工作，组织开展了机关干部下访活动，化解了大量的社会矛盾，促进了社会稳定。

(七)科技教育稳步发展，各项社会事业全面进步。坚持科教兴市战略，组织实施科技计划项目325项，研究开发新技术、新工艺478项，开发新产品68个，产生经济效益18.8亿元。加强教育工作，"两基"成果得到巩固，中小学危房改造步伐加快，办学条件得到改善，高中教育发展迅速，职业教育、成人教育继续发展，黄淮学院申办取得成功。医疗卫生事业快速发展，疾病控制和医疗救治体系建设得到加强，艾滋病防治救助和帮扶工作成效显著。高度重视人口和计划生育工作，人口自然增长率5.01‰，低于全省平均水平。广播电视、文化艺术、新闻出版事业不断进步，群众体育和竞技体育健康发展，成功举办了市第一届运动会。环保工作力度加大，环境质量得到改善。深入开展拥军优属、拥政爱民和军民共建活动，军政军民关系得到巩固，民兵"三带一为"活动成效显著。认真落实民族宗教政策，民族宗教工作深入开展。统计、人事、审计、气象、人防、史志、档案、侨务等各方面都取得了新的成绩。

各位代表，2004年是我市经济社会快速发展、人民群众得到更多实惠的一年。这些成绩的取得，是省委、省政府和市委正确领导的结果，是全市人民团结奋斗、辛勤努力的结果。在此，我代表市人民政府向工作在全市各条战线的广大工人、农民、知识分子、干部、驻驻部队指战员、武警官兵、政法干警和社会各界人士致以崇高的敬意!向所有关心支持政府工作的各位人大代表、政协委员和民主党派、工商联、无党派人士表示衷心的感谢!

在肯定成绩的同时，我们也清醒地看到，当前我市经济社会发展中还存在不少矛盾和问题。主要是:工业基础薄弱，总量小，对经济发展的支撑作用不强;农业主导国民经济增长的特征比较明显，农业产业化水平不高;存贷比例不协调，信贷资金投放偏少，固定资产投资总量较低，经济发展后劲不足;财政收入结构不合理，收支矛盾依然突出;群众关心的一些热点、难点问题还没有得到很好解决;发展环境仍不宽松，在转变政府职能、提高工作效率方面还存在不少问题等等。对此，我们一定高度重视，在今后的工作中认真加以解决。

折叠编辑本段2005年政府工作的目标和主要任务

今年是"十五"计划的最后一年，也是实现本届政府任期目标的关键一年。综合分析当前形势，我们虽然存在一些困难和问题，但也面临着难得的发展机遇。今年中央出台了1号文件，国家继续加大对"三农"特别是粮食主产区的投入和扶持力度，将进一步调动农民生产积极性，促进农村经济快速发展;国家从政策、资金、重大项目布局等方面积极支持中部崛起，沿海发达地区的产业加快向中西部地区转移，有利于我们发挥区位优势和资源优势，加快产业结构转型升级。从省内情况看，全省经济处于一个新的战略起点，统筹区域发展的能力显著增强，有利于我们农业大市的发展。从我市情况看，经过多年的发展，综合实力不断增强，特别是近些年实施了一批工业和基础设施项目，今年将形成新的投资高峰，对全市经济发展必将产生巨大的拉动作用。面对机遇和挑战，只要我们切实增强危机感和紧迫感，抢抓机遇，开拓进取，务实苦干，就一定能够开创各项工作新局面!

今年政府工作的总体要求是:〖HTH〗坚持以邓小平理论和"三个代表"重要思想为指导，全面贯彻落实党的十六大、十六届三中、四中全会及中央经济工作会议、省委工作会议和市委一届七次全会精神，坚持以科学发展观总揽全局，以经济建设为中心，围绕全面建设小康社会和平安崛起的目标，深入开展"三项建设"，强力推进改革开放，调整优化经济结构，加快工业化、城镇化、农业现代化进程，改善人民生活，发展社会事业，促进经济社会全面协调可持续发展。

今年经济和社会发展的主要预期目标是:全市生产总值增长11%，全社会固定资产投资增长25%，社会消费品零售总额增长12%，地方财政一般预算收入增长11%，外贸出口增长16%，外商直接投资增长25%，城镇居民可支配收入增长8%，农民人均纯收入增长5%，城镇新增就业人员3.5万人，城镇登记失业率控制在4.5%以内，人口自然增长率控制在6‰以内。实现上述目标，要重点抓好以下七个方面的工作。

(一)加大重点项目建设力度，大力发展工业经济

继续开展"项目建设年"活动。今年计划安排重点建设项目93个，总投资289.36亿元，年度投资92.08亿元。其中计划竣工项目37个，年度投资26.4亿元;续建项目27个，年度投资58.93亿元;计划新开工项目17个，年度投资6.63亿元;前期项目12个。对于这些重点项目，要千方百计加快工作进度，确保计划竣工项目按时竣工投产，续建项目完成年度投资进度，计划新开工项目如期开工建设。抓住国家和省加大对"三农"等经济社会发展薄弱环节投入的机遇，围绕现有项目、产业和资源优势，抓紧筛选、论证、储备一批大项目和相关配套项目，充实项目库，积极跑省赴京，搞好衔接，争取更多的项目批复立项、获得资金支持。搭建银企合作平台，通过银企座谈会、项目推介会等多种形式，促进银企沟通，争取信贷支持。注重创新融资方式，吸纳外来投资，启动民间资本，鼓励企业通过资本运作方式筹措建设资金。完善市领导分包重点项目责任制和项目建设进度月报制，加大督查力度，定期跟踪问效，及时通报进度，推进项目落实。加强项目管理，落实好项目法人责任制、工程质量终身责任制和建设工程监理制，确保项目建设进度和质量。

发展工业是建设小康、缩小差距的必由之路，也是必然选择。必须牢固树立工业兴市意识，把领导力量向工业倾斜，工作重点向工业转移，突出抓好工业项目建设，进一步营造大上工业项目、大干工业的良好氛围。今年共安排重点工业项目48个，占重点项目总数的51.6%，总投资141.3亿元，年度投资49.2亿元。天方药业技改、骏马化工新建扩建、华骏工业园二期技改、蓝天集团年产30万吨甲醇等25个计划竣工项目，确保年内竣工投产;蓝天集团2×35万千瓦燃气电厂、古城电厂2×30万千瓦净化燃烧机组、吴桂桥煤矿等8个续建项目，确保完成年度投资计划;骏马化工年产2万吨甲胺、河南汇通集团驻马店肉食品公司生猪屠宰等10个计划开工项目，力争按期开工建设;做好豫龙水泥二期扩建、华润电力煤田综合开发等5个项目的前期工作，积极创造开工条件。

继续推进工业结构调整。依托资源优势，重点抓好农畜产品和矿产资源精深加工，拉长产业链条。积极扶持高新技术企业，扩大高新技术产业规模。加大技术改造力度，落实技术改造项目，推进传统产业、产品升级换代。支持优势产业优化重组，引导生产要素向支柱产业聚集，促使天方药业、骏马化工、华骏车辆、蓝天集团等一批骨干企业向大企业集团发展，逐步把我市建设成为豫南较大的化工基地、制药基地、能源基地、建材基地和食品加工基地。加强工业运行监测，定期研究解决工业运行中存在的矛盾和问题。抓好工业运行调节，做好煤炭、电力、运力、资金等方面的供需衔接和协调。重视扭亏增盈工作，引导企业加强内部管理，坚决遏制亏损企业亏损额上升势头，促进工业经济快速发展。

(二)切实加强"三农"工作，推进县域经济发展

坚持把解决"三农"问题作为各项工作的重中之重，围绕农业增效、农民增收和农村发展，强化政策支持，深化农村改革，加大资金投入，加快结构调整，促进农村经济社会全面发展，推动县域经济上台阶。

加大政策扶持力度。采取更加有力的措施，保证行之有效的支农政策落到实处，充分调动农民生产积极性。全面落实免征农业税政策，继续实行对种粮农民的直接补贴，增加良种补贴和大型农机具购置补贴。利用好国家"三奖一补"政策，积极争取奖励和补助资金，缓解县乡财政困难。加大对加重农民负担案件的查处力度，暂不出台"一事一议"筹资政策，确保减轻农民负担各项措施落实。

提高农业综合生产能力。实行严格的耕地保护制度，利用好国家加大对产粮大县扶持的机遇，积极争取中央和省支农资金，抓好一批优质粮食产业工程项目建设。整合农业综合开发等专项资金，建设高标准基本农田。组织实施"科技入户工程"，扶持科技示范户，提高农民科学种田水平。加大科技投入，加强农业技术推广体系、农产品标准质量认证检验检测体系和动物防疫、畜产品质量安全体系建设，推进农畜产品的标准化生产。

加强农业和农村基础设施建设。加大农田水利、中低产田改造、节水农业及灌区配套建设力度，加快农业综合开发项目的实施。积极争取中央和省财政专项补助资金，支持农户投工投劳开展小型农田水利设施建设。抓好小洪河治理、老王坡滞洪区安全等工程建设，启动农村安全饮水工程，做好平原重点湖洼地治理工程和薄山水库、铜山湖除险加固工程等项目的前期工作，确保全年新增有效灌溉面积15万亩，新增旱涝保收田10万亩，治理水土流失面积40平方公里。充分利用村村通优惠政策，积极争取省定项目，加快乡村道路建设步伐。继续抓好荒山绿化和成片造林，加大平原绿化高级达标推进力度，完善提高绿色通道建设，改善农业生态环境。

积极推进农业和农村经济结构调整。继续抓好全市优质小麦基地建设，优化品种，提高品质，扩大生产规模。抓好特色农业生产，集中连片发展优质、高效、生态和无公害农产品，形成各具特色的农业产业带。突出发展节粮型畜牧业，加快畜禽养殖小区建设。大力发展奶业生产，逐步建设一批奶源基地。重视优势农产品原产地注册工作，实施"名牌战略"，提高农畜产品市场竞争力。集中使用扶持农业产业化的资金，培育农畜产品精深加工龙头企业，重点支持河南汇通集团驻马店肉食品公司、天乳奶业和上蔡大程、西平丰盛、遂平益康等加工企业，加强与国内大型食品加工企业的合作，争取在我市建立分厂或加工基地，提高农畜产品的加工转化能力。鼓励龙头企业以多种利益联结方式，带动基地和农户发展，提高农业产业化水平。

努力提升劳务经济。增加对农民职业技能培训的投入，提高培训的实用性，鼓励社会力量参与农民职业技能培训。加强对劳务输出的服务和管理，健全劳务输出信息网络，继续引导农村劳动力合理有序转移。充分发挥劳动力资源优势，扩大劳务输出规模，打造劳务经济品牌，力争全市农村劳务输出保持在160万人以上。

认真落实支持县域经济发展的各项政策，积极争取县域经济发展专项资金，重点支持环保、旅游等基础设施和特色工业项目建设。利用好城镇化建设引导资金，支持重点县、镇城镇化建设。加强城乡市场体系建设，规划建设一批县域综合农业生产资料市场。突出抓好民营经济，完善政策措施，抓好政策落实。引导民间资本，投入工业项目，支持民营企业参与能源、交通、城市公共设施建设。加强服务和监督，确保民营企业在土地征用、投资立项、信贷支持等方面与其他企业享受同等待遇。引导商业银行加大对民营企业的信贷支持，鼓励民营企业建立商业性或互助性信用担保体系，拓宽民营企业的融资渠道。指导民营企业加强内部管理，规范经营行为，提高产业层次，加快建立现代企业制度，增强抵御市场风险的能力。

(三)深化经济体制改革，提高对外开放水平。

继续搞好国有企业改革。采取有效形式，吸引外资和民间资本参与国有企业股份制改造，逐步实现股权多元化，大力发展混合所有制经济。着力培育和发展一批具有较强市场竞争力的大企业和企业集团，优化资源配置，壮大企业实力。完善公司法人治理结构，引导企业深化人事、劳动和分配制度改革，增强企业发展活力。以剥离不良资产为切入点，加大商贸、城建、水利、粮食、医药、供销、交通等系统直属企业以及经营性事业单位的改革力度，强化措施，落实责任，力争年内改制面达到50%以上。推进粮食购销体制改革，转换企业经营机制，搞好人员分流安置，力争完成国有粮食购销企业改制任务。加快农村信用社改革进度，继续抓好增资扩股和清收不良贷款工作，确保如期拿到专项央行票据并实现兑付。完善农村信用社法人治理结构，创新经营机制，增强支持农村经济发展的能力。

积极推进财税体制和其他领域的改革。完善财政收支管理，继续推进部门预算、国库集中收付、政府采购、乡财县管和"收支两条线"管理等改革。强化税收征管，确保财政收入稳步增长。调整和优化支出结构，严格控制一般性支出，保证重点支出，充分发挥公共财政的作用。加强对财政支出的审计工作，提高财政资金使用效率。进一步推进农村义务教育体制、乡镇机构和县乡公共财政体制改革，避免发生新的乡村不良债务，防止农民负担反弹。加大事业单位管理体制、用人制度、分配制度改革力度，增强事业单位发展活力。进一步完善招投标制，加强对工程建设、政府采购、药品采购等招投标工作的监管，严格运作程序，防止暗箱操作。放开基础设施、公用事业建设经营市场，逐步建立投资多元化、经营企业化、管理法制化的城市公用事业建管体系。继续抓好文化、教育、卫生等社会事业领域的改革。

努力提高对外开放水平。坚持以东西合作为载体，抓住国际、国内产业加快转移的机遇，强化措施，加大力度，大力发展开放型经济。围绕我市资源和产业优势，选择重点地区，搞好项目对接，开展有针对性的招商引资活动，提高招商引资实效。注重发挥企业的主体作用，积极筛选一批好项目，优先推出一批优势企业对外招商。培育招商中介组织，采取以商招商、代理招商、委托招商等形式，多渠道引进和利用国内外资金，扩大引资规模。抓好签约项目履约工作，强化责任，搞好跟踪问效，提高项目履约率和资金到位率。引导合作项目向园区集中，提高产业的集聚度。做好今年东西合作会的筹备工作，创新办会路子，加强项目衔接，确保取得更加丰硕的成果。加强对高新区工作的领导，发挥其在全市招商引资工作中的窗口、示范和带动作用。不断扩大外贸出口，鼓励各类所有制企业开展出口业务，培育新的出口增长点。继续支持天方药业、华中正大、康茂、荣光鞋业等出口创汇企业做大做强。加快农畜产品出口基地建设，扩大产品出口规模。做好外派劳务工作，增加外派劳务的数量。

大力优化经济环境。完善和落实招商引资优惠政策，增强对外招商的吸引力，切实做到引得来、留得住、发展好;规范部门执法行为，从严控制对企业的检查评比，全面落实服务承诺制、限时办结制和一站式审批、一条龙服务;加大环境治理力度，严厉打击强装强卸、强买强卖、欺行霸市和"四乱"行为，对侵害企业利益、干扰企业经营、阻挠企业施工的典型案件，发现一起，查处一起，曝光一起，着力营造政策宽松、办事快捷、服务周到的良好发展环境。

(四)积极推进城市发展，加快城镇化步伐

高标准做好城镇规划。以创建"园林城市、卫生城市"为目标，以新区规划和旧城区人居环境改善为重点，全面完成城市近期建设规划以及城市道路、防洪、环卫设施、商业网点等专项规划，不断提高老城区控规覆盖率。坚持以人为本，提高品位，完成城市主干道、市区主要出入口和公共场所的景观规划。以县城和中心镇为重点，搞好城镇规划。加强规划管理，规范审批程序，加大执法力度，维护规划的严肃性和权威性。

加强城市建设和管理。突出抓好中心城区建设，加快会展中心、天中广场、开源河等新城区项目建设，全面实施行政区北迁工程。推进前进路北段新建、练江路改造建设步伐，抓好春晓街东段等旧城改造项目建设;确保西气东输地方支线、9县1区城市管网、热电厂集中供热等5个续建项目，完成年度投资计划。加快金色置地、建业绿色家园、通达小区房地产项目建设进度，年内完成主体工程;完成市区居民生活供水"明渠改暗管"工程，做好市第二水厂建设前期工作;抓好新一代雷达中心和科技馆建设，使其成为城市标志性建筑。加强城市管理，以治理脏、乱、差为重点，继续开展城市环境综合整治活动，明确市区分级管理责任，完善长效管理机制，增强市民文明意识，不断提高城市管理水平。完善旅游基础设施，抓好重点景区建设，搞好红色旅游开发，加快旅游业发展。

搞活城市经营。按照政府引导、市场运作的思路，树立经营城市理念，组建城市建设投资开发有限公司，搭建城市建设招商引资平台，引导社会资金参与城镇基础设施建设，走以城养城、以城建城的路子。搞好城市土地资源的市场化运作，对于商业、旅游、娱乐、商品住宅等经营性用地，一律实行招标、拍卖或挂牌方式出让。对于城市道路、桥梁、广场等，通过拍卖冠名权、经营权、开发权等形式，搞好经营。对于城市供水、供热、供气和污水、垃圾处理等经营性公用事业，实行统一规划开发、多种形式经营，提高城市经营效益。

抓好公路交通项目建设。发挥大交通在拉大城市框架和城镇化进程中的作用，努力构建以市区为中心、连接城乡的交通网络。加快确平路、驻信高速两条连接线及20条县乡公路建设进度，确保建成通车。阿深高速正平段、上武高速泌阳段、新阳高速等7个续建项目，完成路基土方、涵洞通道和桥梁工程。做好君二路(汝南老君庙-确山县城)、张南路(平舆和店-平舆县城)、商桐路(确山竹沟-驻南交界)等5个前期项目准备工作，力争年内开工建设。

加快小城镇建设步伐。制定和实施促进小城镇建设的引导性政策和措施，吸引各类生产要素向小城镇聚集。引导小城镇立足实际，培育支柱产业，以产业聚集带动城镇发展壮大。搞好小城镇规划，加强基础设施建设，完善城镇功能，发挥其对农村的辐射带动作用，逐步形成城乡互动、以城带乡的良好局面。

(五)坚持以人为本，切实改善人民群众的生活

认真做好就业再就业工作。大力发展劳动密集型企业和第三产业，千方百计扩大就业门路，拓宽就业领域。加大督查协调力度，落实好小额贷款、税费减免等再就业扶持政策。深入开展帮扶活动，搞好就业援助和就业服务，优先安排"4050"人员等就业困难对象进入政府开发的公益性岗位，确保全年新增就业人员3.5万人，下岗失业人员实现再就业1万人。

加强社会保障体系建设。继续做好"两个确保"工作，规范"低保"标准和范围，做到应保尽保。扩大城镇职工社会保险覆盖面，加大保险费征缴力度，提高征缴率。做好国有企业下岗职工出中心工作，实现国有企业下岗职工基本生活保障向失业保险并轨。切实做好救灾工作，完善农村五保户、特困户救助和各项敬老优待政策，保障农村困难群众的基本生活。

重视解决关系人民群众切身利益的问题。抓好扶贫开发工作，努力实现8万贫困人口脱贫任务。积极争取国家、省财政专项资金，解决好农村群众的饮水困难和饮水安全问题。加大清理拖欠工程款和农民工工资力度，努力完成年度清欠目标。规范土地征用、城镇拆迁工作，解决好失地农民和城镇拆迁户的补偿安置，保护城乡居民的合法权益。实施食品药品放心工程，严厉打击制售假冒伪劣食品、药品等违法犯罪活动，维护人民群众生命安全。

(六)统筹经济社会发展，推进社会事业全面进步

加强科技教育工作。积极开展与大专院校、科研院所、高科技企业的合作，做好先进实用科技成果的引进、推广和应用。加大投入，组织实施一批科技攻关项目，抓好农业科技示范园区建设，充分发挥科技对经济的支撑和带动作用。重视人才工作，完善政策措施，开发人力资源，创造吸引人才、留住人才的良好环境。坚持把教育放在优先发展的战略地位。加强农村基础教育，巩固"两基"成果;实施好农村中小学危房改造工程，力争提前一年完成D级危房改造任务;抓好高中建设与发展，扩大高中教育规模;大力发展职业教育，着力为经济建设培养更多的适用技术人才;加大对黄淮学院支持力度，加快高等教育发展步伐。

加强疾病预防控制、医疗救治、卫生监督三大体系建设，促进卫生事业发展。加大艾滋病防治和救助力度，完善综合防治措施，健全防治救助长效机制，切实控制艾滋病的传播和蔓延。搞好乡镇卫生院建设，尽快完成乡镇卫生院上划县级管理工作。实施文化精品工程，繁荣文学艺术、广播电视、新闻出版事业。开工建设市图书馆，抓好文化市场管理，开展广播电视"农村服务年"活动，推进文化产业发展。加强基层群众文化阵地建设，积极开展各类群众性文化活动和全民健身活动。加强国防教育，做好国防动员、人民防空和民兵预备役工作，落实优抚及退伍安置政策，巩固"双拥"成果。依法保护妇女、儿童、未成年人和残疾人的合法权益。继续做好审计、统计、人事、外事侨务、史志、气象、红十字会等工作。

实施可持续发展战略。坚持计划生育国策地位和现行生育政策不动摇，落实一把手负责制和一票否决制，稳定低生育水平。加强土地、水、森林、矿产等资源管理，提高资源利用效能。以水污染防治为重点，加大环境保护力度，开工建设市区垃圾处理厂、市污水处理厂管网配套设施和西平县污水处理厂，做好污水处理费征缴工作。积极推行清洁生产，落实各项污染防治措施，努力改善生态环境。

(七)推进精神文明和民主法制建设，构建和谐社会

加强精神文明建设。认真贯彻《公民道德建设实施纲要》，全面加强社会公德、职业道德和家庭美德教育，抓好青少年的思想道德教育，提高全民思想道德素质。围绕创建省级文明城市，深入开展文明单位、文明行业、文明社区、文明村镇创建活动。加强诚信教育，推动和促进全市信用体系建设。积极开展科技、文化、卫生"三下乡"活动，破除封建迷信，深化殡葬改革，倡导文明健康的生活方式，营造文明和谐的社会环境。

推进民主与法制建设。认真执行人大及其常委会的决议、决定，坚持重大事项报告制度，支持人大代表依法行使职权。加强与人民政协的协商，自觉接受人民政协民主监督。认真办理人大代表议案、建议和政协提案。广泛听取各民主党派、工商联和无党派人士的意见，充分发挥工会、共青团、妇联等人民团体的桥梁纽带作用。认真贯彻党的民族宗教政策，继续做好民族宗教工作。全面推进政务、厂务、村务公开，完善村民自治和企业民主管理制度，做好第五届村民委员会换届选举工作。加强法制宣传教育，推进依法治市，不断提高全社会的法制意识。

继续深入开展"平安建设年"活动。加强基层基础工作，始终保持严打态势，严厉打击黑恶势力犯罪、严重暴力犯罪、多发性侵财犯罪，依法打击等邪教组织，全面落实社会治安综合治理措施，继续争创全省社会治安综合治理工作先进市。高度重视安全生产工作，狠抓安全生产措施的落实，加强安全生产监督管理，加大事故责任的查处和追究力度，严防各类重大事故发生。认真贯彻落实国务院《信访条例》，重视人民群众来信来访，切实解决好人民群众反映强烈的问题;坚持依法信访，规范信访秩序，妥善处置各类群体性事件，维护社会大局稳定。

折叠编辑本段努力提高政府施政能力

今年加快发展的任务非常艰巨，要全面完成各项目标，必须坚持以经济建设为中心，转变职能，改进作风，把政府施政能力提高到一个新水平。

折叠牢固树立中心意识，突出发展主题

始终把发展作为政府工作的第一要务，坚持全部工作以经济建设为中心，切实做到一切思路围绕经济发展去谋划，一切措施围绕经济发展来制定，一切工作围绕经济发展去落实。强化发展意识，完善目标考评办法，明确任务，加压增责，充分激发各级政府、各部门加快发展的主动性、创造性，努力形成全市上下争先恐后、竞相发展的良好局面。继续解放思想,深化改革，坚决冲破一切妨碍发展的思想观念，坚决改变一切束缚发展的做法和规定，坚决革除一切影响发展的体制弊端，充分调动各种积极因素，集中全市人民的智慧和力量，聚精会神搞建设，一心一意谋发展，进一步营造"以发展论英雄，以项目论政绩，以招商引资论本领"的良好氛围。

折叠转变政府职能，提高行政效率

按照市场经济的要求，切实把政府职能转变到为市场主体服务和创造良好的发展环境上来，努力建设服务型政府。改进经济管理的方式和方法，善于运用经济手段，处理好速度与质量、结构、效益之间的关系，着力提高经济增长的质量和效益。坚持以人为本，把更多的财力、物力等资源用于提供公共产品，搞好公共服务。完善市场体系，加强市场监管，继续整治市场经济秩序，严厉打击破坏和扰乱市场秩序的行为，切实为各类市场主体创造公平竞争的环境。依法管理和规范社会组织、社会事务，协调好各种利益关系，促进社会公平和正义。建立健全重大事项社会公示、社会听证与专家咨询制度，深化县乡两级政务公开，全面推行市级政务公开，增强政府决策的公众参与度，推进决策民主化和科学化。积极推进电子政务，完善行政服务大厅功能，改进服务方式，提高服务效率。

折叠坚持依法行政，规范行政行为

树立依法行政观念，严格按照法定权限和程序行使权力，履行职责。加强行政执法队伍建设，落实行政执法人员持证上岗和亮证执法制度，积极推行行政执法部门主要岗位轮岗制，规范行政执法行为。深入贯彻《行政许可法》，继续深化行政审批制度改革，依法规范行政许可行为。严格实行执法责任制、错案追究制和行政赔偿制，认真解决有法不依、执法不公和多头执法、执法扰民等群众反映强烈的问题。进一步加强对行政权力的监督，规范行政行为，做到用权受监督，侵权要赔偿，违法受制裁。

折叠改进工作作风，务求工作实效

深入开展"作风建设年"活动，大兴求真务实之风。牢固树立正确的政绩观，坚持人民利益高于一切，坚决杜绝劳民伤财的"政绩工程"、"形象工程"。下大力气精简会议和文件，改进会风和文风，最大限度减少应酬和不必要的事务性活动，拿出更多的时间和精力开展调查研究，深入了解社情民意，认真解决实际问题，多为群众办实事、办好事。坚持和完善行风评议制度，推进机关作风转变，增强服务意识，提高办事效率。强化工作责任，加强督促检查、跟踪问效，反对敷衍塞责、推诿扯皮，确保政令畅通和工作落实。坚持廉洁从政，从严治政，认真落实党风廉政建设责任制，努力从源头上预防和治理腐败，以廉政建设的实际行动取信于民。

各位代表，全面建设小康社会、实现天中平安崛起，是驻马店830万人民的迫切愿望，我们面临的任务艰巨而繁重。让我们高举邓小平理论和"三个代表"重要思想的伟大旗帜，在省委、省政府和市委的领导下，在市人大、市政协监督支持下，紧紧依靠全市人民，团结奋进，开拓创新，扎实工作，为振兴全市经济、构建和谐社会而努力奋斗!
